# Quantitative Job Insecurity and Well-Being: Testing the Mediating Role of Hindrance and Challenge Appraisals

**DOI:** 10.3389/fpsyg.2018.02776

**Published:** 2019-01-25

**Authors:** Morteza Charkhabi

**Affiliations:** Department of Psychology, National Research University Higher School of Economics, Moscow, Russia

**Keywords:** job insecurity, well-being, hindrance appraisals, challenge appraisals, job satisfaction, emotional exhaustion

## Abstract

The aim of this study is to determine the extent to which cognitive appraisals of job insecurity may mediate the link between job insecurity and well-being among employees. According to cognitive appraisal theory, the two cognitive appraisals of job insecurity, hindrance vs. challenge appraisals, were integrated into a conceptual model and examined as the mediators of job insecurity-wellbeing association. Well-being related outcomes were job satisfaction and emotional exhaustion. Hypotheses were tested using a total sample of 306 hospital staff^1^. Respondents from diverse departments of this hospital were recruited and completed scales on quantitative job insecurity, hindrance vs. challenge appraisals of job insecurity, job satisfaction, and emotional exhaustion. Results indicated that hindrance appraisals of job insecurity mediated the association between job insecurity and emotional exhaustion. Challenge appraisals did not mediate the job insecurity-well-being association. In all, only one out of four mediation paths was found significant. As a result, employees hindered by job insecurity are more likely to be emotionally exhausted.

## Introduction

Job insecurity, as a worldwide chronic work stressor, has attracted increased research attention since the beginning of the 21st century (i.e., McDonough, 2000; Probst, 2008; De Witte et al., 2015). Many studies have explored the detrimental effects of this stressor on employees and organizations (i.e., De Witte, 2005; Jiang and Probst, 2015). Job insecurity is mainly defined as an overall concern of an employee about the continued existence of the job in the future (Vander Elst et al., 2010b). Recent studies show that the current economic climate, instability in employment conditions, and large-scale structural changes may initiate or intensify the perception of job insecurity among employees (De Witte et al., 2015; Schaufeli, 2016).

Job insecurity is a two-dimensional construct that is classically divided into quantitative and qualitative job insecurity. Quantitative job insecurity is related to the concern of employees about the continued existence of their job in the future (Vander Elst et al., 2010b), while qualitative job insecurity is related to the concern of employees about the extent to which their job features may unfavorably change (e.g., Hellgren et al., 1999; De Witte, 2005). Although studies show that both types of job insecurity can lead to negative outcomes, the focus of this study will be on quantitative job insecurity. Studies show that an increase in job insecurity is associated with an increase in various work-related strains. At the individual level, job insecurity is related to negative outcomes such as psychological distress and emotional exhaustion (e.g., Cheng and Chan, 2008; Piccoli and De Witte, 2015). At the organizational level, job insecurity is associated with a lower job satisfaction and job commitment and a higher turnover intention (Furåker and Berglund, 2013; Lim et al., 2014; Hamad et al., 2015; Wang et al., 2015). Although studying the link between job insecurity and different outcomes has gained a lot of research attention, there has been lesser attention to the underlying mechanisms that can explain this link. The current study intends to expand knowledge on the theoretical explanations of the link between job insecurity and well-being related outcomes through testing the role cognitive appraisals as the mediators.

The focus of this article is on two possible cognitive mediators, hindrance and challenge appraisals, which have the potential to mediate the detrimental effects of job insecurity on the outcomes. Consistent with previous studies (e.g., Sverke et al., 2002; Zheng et al., 2014; Öztürk et al., 2017), job satisfaction and emotional exhaustion were used as the two popular well-being related outcomes of job insecurity. Job satisfaction is defined as the degree to which employees have a positive affective orientation toward employment by the organization (Price, 1997). Emotional exhaustion is a chronic state of physical and emotional depletion that results from excessive job, personal demands, and/or continuous stress (Maslach and Leiter, 2008; Piccoli and De Witte, 2015).

### Contributions of the Current Study

Studies show that job insecurity is a subjective construct which may vary from an individual to another (Sverke et al., 2002; De Witte, 2005). It has been found that personality and attitudinal factors can influence the link between job insecurity and well-being related outcomes (i.e., Vander Elst et al., 2014b; Piccoli and De Witte, 2015) but no study thus far considered the extent to which the employees’ cognitive appraisals of job insecurity may affect this association. Based on appraisal theory (Lazarus and Folkman, 1984), we distinguish two types of appraisals, hindrance vs. challenge appraisals, as two cognitive mediators which may potentially explain the job insecurity-well-being association. Hindrance appraisals are related to the appraisal of threats as “harms or losses” that have not yet taken place but are anticipated to occur. Challenge appraisals are associated with the appraisal of threats as “gains or growths” in a situation and are recognized as the facilitator of personal growth and goal attainment at the individual level (Barsky et al., 2011). Empirically testing the mediating role of these two appraisals in the job insecurity-well-being association is the main aim of this study. Appraisal theory (Lazarus and Folkman, 1984) will be used as main theory and conservation of resources theory (Hobfoll, 1989) as a supplementary theory to make predictions regarding the mediating role of both appraisals.

### Hindrance vs. Challenge Appraisals: Mediators?

Many current researchers (e.g., Sverke et al., 2002; Probst et al., 2013; De Witte et al., 2015, 2016) have shared the job insecurity-strain view to explain the way that job insecurity affect well-being related outcomes. Although this view has widely been corroborated (e.g., De Witte, 2005; Cheng and Chan, 2008; Schaufeli, 2016), still not much is known about the role of cognitive appraisals of job insecurity in this link. Actual job insecurity and appraisal of job insecurity are two distinct constructs. As defined, job insecurity is the concern of employees about the continued existence of the job in the future and it is considered a chronic work stressor (i.e., De Witte et al., 2015). The appraisal of an employee of job insecurity can be negative (threatening) or positive (challenging). According to appraisal theory (Lazarus and Folkman, 1984), characteristics of a situation and personal resources of an individual result in primary and secondary appraisals of a stressor/event. Primary appraisal is thought to determine if an event or aspect of the environment is perceived as a hindrance or a challenge, and is known as one of the main psychological mechanisms linking stressors to strains (Webster et al., 2011). An individual with hindrance appraisals is expected to focus on the negative aspects of a stressor (i.e., harms or losses) by overestimating negative aspects and an individual with challenge appraisals is assumed to concentrate on the positive aspects of a stressor (e.g., gains or growths) by overestimating positive aspects. Secondary appraisals involve evaluating one’s capacity to cope/deal with a situation. This concerns evaluations of factors such as the personal resources of individuals to regulate a stressful situation/event (Weiss et al., 1999; Barsky et al., 2011; Vander Elst et al., 2014a). The implication of this categorization (hindrance vs. challenge) is that different individuals can interpret the same stressor in both ways (Hobfoll, 1989) and that one person can even appraise a stressor as a hindrance and challenge simultaneously (Lazarus and Folkman, 1984). For example, studies show that workload, as a popular work-related stressor, can be appraised as a challenge (e.g., Marsh, 2001) or a hindrance (e.g., Cavanaugh et al., 2000). However, this distinction still seems to be unclear for job insecurity, since the criteria of studies for considering job insecurity as a hindrance (i.e., De Witte et al., 2016) or challenge (i.e., Glavin and Schieman, 2014) has been based on its association with the outcomes of job insecurity rather than on the appraisal of job insecurity itself. Since scholars thus far have not been analyzing whether job insecurity as such (regardless of its relationship with outcomes) is appraised as a challenge or a hindrance, this research intends to fill this research gap by the direct measurement of hindrance and challenge appraisals of job insecurity. Furthermore, no study could be found to show how cognitive appraisals of job insecurity influence the association between job insecurity and well-being. As noted earlier, a hindrance appraisal of job insecurity may frustrate or pose a threat in reaching one’s goals (obstacles that could be hardly overcome). A challenge appraisal, on the contrary, may facilitate goal achievement for individuals through being hard working to secure their job (obstacles that you think you can easily overcome) (Webster et al., 2011). By definition, hindrance appraisals may constrain or interfere with a person’s perceived ability to fulfill a job demand or deal with a work stressor. On the other hand, challenge appraisals might be positively related to a person’s perceived ability to fulfill a job demand or deal with a situational stressor such as job insecurity. As such, this study aims to fill this research gap by examining the mediating role of hindrance and challenge appraisals of job insecurity. This leads to the following hypotheses:

Hypothesis 1. Hindrance appraisals of job insecurity mediate the association between: (a) job insecurity and job satisfaction, and (b) job insecurity and emotional exhaustion.

Hypothesis 2. Challenge appraisals of job insecurity mediate the association between: (a) job insecurity and job satisfaction; (b) job insecurity and emotional exhaustion.

**FIGURE 1:**
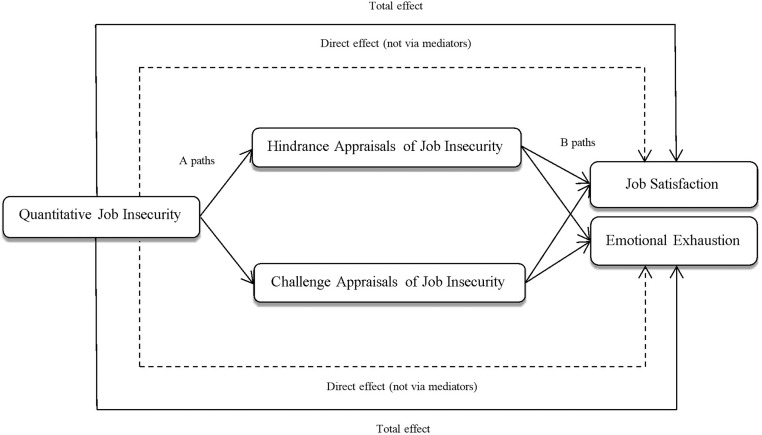
A proposed model of the direct and in direct paths.

Figure 1 displays the proposed model of the relationship between the research variables. In this study, hypotheses are tested using two separate mediation paths.

## Materials and Methods

### Participants

In order to test our hypotheses, surveys were administered to 368 employees from Iran. These employees were sampled from a large public hospital located in Iran. In this sample, the response rate was approximately 83% (*N* = 306). Participants included 58.82% female and 41.18% male employees from different departments of this hospital. The mean age of the participants was 33.5 years (*SD* = 7.83), and their mean work experience was 9.4 years (*SD* = 7.95). 86.6% of the participants were administrative employees (e.g., secretary, IT operator, shift planners, supervisors) and 13.4% were professional employees (e.g., nurses, medical assistants, and patient transferor, laboratory pathologist, radiologist). 20.3% had a permanent contract whereas 79.7% had a temporary contract. Finally, 88.57% of respondents had received at least a college diploma, while 11.43% had a high school diploma or less.

### Procedure

The research staff distributed informed consent letters aligned with surveys among participants explaining the anonymous nature of the data collection and their rights as research participants to participate/withdraw. The participants provided written and informed consent during their working hours. To ensure that participants were comfortable to respond to the questions, they were informed that only members of the research team would have access to the data. Supervisors were not present when the data were collected.

### Measures

#### Job Insecurity

Perceived job insecurity was measured by the four-item job insecurity scale (JIS) developed by De Witte (2000) and validated by Vander Elst et al. (2014a). This scale is a global job insecurity measure that corresponds to our conceptualization of job insecurity: it includes items that refer to the threat or the possibility of losing a job, as well as an item that refers to the worries associated with job loss. An example item is “*I think I might lose my job in the near future.*” Respondents were asked to rate the items on a scale from 1 (totally disagree) to 5 (totally agree). Responses were scored such that higher numbers reflect higher job insecurity. The reliability (Cronbach alpha) was 0.77.

#### Emotional Exhaustion

The Maslach Burnout Inventory-General Survey (MBI-GS) (Maslach et al., 1996) was used to measure emotional exhaustion. The MBI-GS has three sub-scales; however, only the five items of the emotional exhaustion subscale was used. An example item is “*I feel used up at the end of the workday.*” Items are scored on a 5-point frequency rating scale ranging from “1” (never) to “5” (daily). High scores reflect higher emotional exhaustion. The reliability (Cronbach alpha) was 0.92.

#### Job Satisfaction

The four-item scale of job satisfaction developed by Price (1997) was used. An example of an item is “*Most days I am enthusiastic about my job.*” Respondents were asked to rate the items on a five-point Likert scale from 1 (totally disagree) to 5 (totally agree). Responses were scored such that higher numbers reflect higher job satisfaction. The reliability (Cronbach alpha) was 0.72.

#### Hindrance vs. Challenge Appraisals of Job Insecurity

These appraisals were measured with a scale initially constructed in Belgium (Peeters, 2014) and its latestet version developed by Charkhabi et al. (2015) in Iran and Italy. This scale included six items, three for hindrance appraisals and three for challenge appraisals. An example of an item of the challenge appraisal component is “*Job insecurity makes me focus on my work so that I can perform well.*” An item example of the hindrance appraisal component is “*Job insecurity undermines my concentration on my job.*” Respondents were asked to rate the items on a scale from 1 (totally disagree) to 5 (totally agree). Responses were scored such that higher scores reflect a higher hindrance or challenge appraisal. The reliability (Cronbach alpha) of the hindrance and challenge components for the Iranian sample were 0.83 and 0.70 respectively.

### Data Analyses

Confirmatory factor analysis (CFA) was used to test the factorial structure of the hindrance vs. challenge appraisals of job insecurity scale using AMOS-21 (Arbuckle, 2005). The CFA was run using the maximum-likelihood method. Because a fit index reflects only a specific aspect of the model fit, a single good value cannot provide enough evidence for a good fit (Kline, 1998; Hu and Bentler, 1999; Vander Elst et al., 2010a). Thus, the goodness-of-fit of the models was estimated by means of several indexes that were interpreted relatively to each other (as suggested by Bollen and Long, 1993; Byrne, 2001): Chi-square statistic (χ2); Comparative fit index (CFI); Tucker-Lewis Index (TLI); Root mean square error of approximation (RMSEA); Standardized Root Mean Square Residual (SRMR); Bayesian Information Criterion (BIC); Akaike’s Information Criterion, single sample cross-validation index (AIC); and (6) Expected Cross-Validation Index (ECVI). For the RMSEA, values smaller than 0.08 indicate good fit (Browne and Cudeck, 1993; Hu and Bentler, 1999; Byrne, 2001). Values greater or equal to 0.90 on the CFI and the TLI indicate good fit (Hoyle, 1995). BIC, AIC, and ECVI are used in comparing models: the model with the smallest value of BIC, AIC, or ECVI should be chosen as the best. Since the Chi-squared statistic is sensitive to the sample size and tests whether the model shows an exact fit to the data, a finding that is rare, it should not be used as a direct indication for the goodness-of-fit of a model (Weston and Gore, 2006). Hence, it was only used to compare competing models (Weston and Gore, 2006).

## Results

### Preliminary Analyses on the “Cognitive Appraisals of Job Insecurity Scale”

**FIGURE 2:**
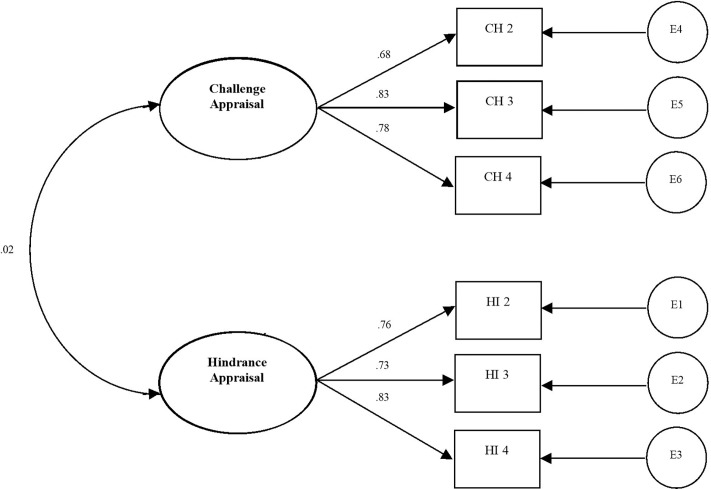
A measurement model of the challenge-hindrance appraisal of job insecurity scale.

Before testing the hypotheses, the factorial structure of the hindrance vs. challenge appraisals of JIS was tested. Four models were tested and compared on the total sample size (*N* = 306) using CFA (see Table 1). At first, the model with seven items loading on one factor (cognitive appraisal) was estimated (Model 1). This model showed bad fit indexes (RMSEA = 0.26, CFI = 0.55, TLI = 0.10) and some very low factor loading (i.e., 0.35). To enhance the model indexes, the first model was revised and substituted with the expected two-dimensional model in which hindrance and challenge appraisals were set as the two correlated latent variables (Model 2). In Model 2, the first factor (challenge appraisal) contained four observed variables and the second factor (hindrance appraisal) three items. Model 2 showed an improvement in all fit indexes (RMSEA = 0.09, CFI = 0.94, TLI = 0.88), but one factor loading (CH1) remained problematic (i.e., 0.34) and was discarded. Therefore, the third model (Model 3) was composed of 6 items and two covariating latent factors (see Figure 2). Fit indexes were very good (RMSEA = 0.05, CFI = 0.98, TLI = 0.96), and standardized factor loadings ranged from 0.69 to 0.81 and were all significantly different from zero. The covariance between the two latent factors was not significant (*r* = -0.08*, p* = 0.065). Model 4 is a model in which the two latent factors did not covariate. The fit indexes were similar to the ones of Model 3 (RMSEA = 0.05, CFI = 0.98, TLI = 0.96). The very similar fit indexes of the two models show that the two models are practically identical and the two dimensions of the appraisal of job insecurity seem not to be necessarily related. However, based on the parsimony principle, the Model 3 was selected as the final model to use for further analyses. We did not need to covariate errors in the final model. The final model, with standardized factor loadings, is illustrated in Figure 2.

**Table 1:** Goodness-of-Fit indexes of challenge-hindrance appraisal of job insecurity scale (*N* = 306).

Models	X^2^	df	CFI	TLI	RMSEA^∗^	SRMR	ΔCFI	ΔRMSEA
Model 1. One factor – 8 items	370.33	20	0.61	0.46	0.24	0.17	–	–
Model 2. Two factors – 8 items	40.09	23	0.96	0.94	0.08	0.04	–0.35	0.16
Model 3. Two factors – 6 items	20.70	8	0.98	0.96	0.07	0.04	–0.02	0.01


*^∗^CI, 95% confidence interval; Note: χ^2^, chi-square goodness of fit statistic; df, degrees of freedom; CFI, Comparative Fit Index; TLI, Tucker Lewis Index; RMSEA, Root-Mean-Square Error of Approximation; SRMR, standardized RMR, root mean square residual.*

### Descriptive Statistics

The descriptive statistics of the scales (means and standard deviations) and the Pearson correlations between the variables are reported in Table 2. In the total sample (*N* = 306), the correlation matrix indicated that job insecurity is negatively correlated with job satisfaction (*r* =-0.218, *p* < 0.001) and positively with emotional exhaustion (*r* = 0.241, *p* < 0.001). In addition, the hindrance appraisal of job insecurity is negatively correlated with job satisfaction (*r* =-0.131, *p* < 0.05) and positively with emotional exhaustion (*r* = 0.269, *p* < 0.001). Also for this sample, there were no significant associations between the challenge appraisals of job insecurity and well-being related outcomes.

**Table 2:** Means, standard deviations, and correlations among the variables (*N =* 306).

Variable	Items	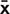	*SD*	1	2	3	4	5
1. Job insecurity	4	2.95	0.886	–				
2. Challenge appraisal	3	2.74	0.930	0.058	–			
3. Hindrance appraisal	3	2.59	0.956	0.298***	0.030	–		
4. Job satisfaction	4	2.65	0.588	–0.218***	0.058	–0.131*	–	
5. Emotional exhaustion	5	3.17	1.09	0.241***	0.053	0.269***	–0.519***	–


*^∗^p < 0.05, ^∗∗^p < 0.01, ^∗∗∗^p < 0.001.*

### Test of the Mediation Effects of Cognitive Appraisals

According to Figure 1, job insecurity was modeled as a predictor, and job satisfaction and emotional exhaustion as the predicted variables. Model 4 of Process program, developed by Hayes (2012), was used to test the mediation paths. Hindrance and challenge appraisals of job insecurity were added as cognitive mediators. In the first phase, the mediation effect of hindrance appraisals of job insecurity between job insecurity and well-being related outcomes was tested. These results are displayed in Table 3. As the table shows job insecurity predicted hindrance appraisals (*β* = 0.30*, p* < 0.001), as expected. Although job insecurity did not predict job satisfaction but it predicted emotional exhaustion (*β* = 0.18*, p* < 0.001). Also, hindrance appraisals of job insecurity did not predict job satisfaction but they predicted emotional exhaustion (*β* = 0.19, *p* < 0.001). The results of the mediation analysis showed that hindrance appraisals of job insecurity did not mediate the association between job insecurity and job satisfaction but they mediated the association between job insecurity and emotional exhaustion (*β* = 0.06*, p* < 0.05).

**Table 3:** Regression results (standardized regression coefficients) predicting the outcomes (*N =* 306).

Effect	*β*	*SE*	*t*	*p*	LLCI	ULCI
Job insecurity to hindrance appraisals	0.301	0.054	5.50	Sig	0.1934	0.4087
Hindrance appraisals on job satisfaction	0.053	0.060	0.883	ns	–0.0651	0.1714
Direct effect of job insecurity on job satisfaction	–0.065	0.060	–1.091	ns	–0.1839	0.0526
Indirect effect of job insecurity on job satisfaction via hindrance appraisals	0.016	0.016	–	ns	–0.0121	0.0524
R-squared mediation effect size	–0.001	0.002	–	ns	–0.0127	0.0013
Hindrance appraisals on emotional exhaustion	0.193	0.057	3.367	Sig	0.0803	0.3061
Direct effect of job insecurity on emotional exhaustion	0.184	0.057	3.212	Sig	0.0714	0.2973
Indirect effect of job insecurity on emotional exhaustion via hindrance appraisals	0.058	0.022	–	Sig	0.0222	0.1097
R-squared mediation effect size	0.027	0.011	–	Sig	0.0113	0.0588

*R*^2^ = 0.09; *F*_(1,304)_ = 30.29, *p* < 0.0000						


*p < 0.05, p < 0.01, p < 0.001.*

In the second phase, the mediation effect of challenge appraisals of job insecurity between job insecurity and those same outcomes was tested. These results are displayed in Table 4. As the table shows job insecurity predicted challenge appraisals (*β* = 0.19, *p* < 0.001), as expected. Although job insecurity did not predict job satisfaction but it predicted emotional exhaustion (*β* = 0.23, *p* < 0.0001). Also, challenge appraisals of job insecurity did not predict job satisfaction and emotional exhaustion. The results of the mediation analysis showed that challenge appraisals of job insecurity did not mediate the association between job insecurity and both job satisfaction and emotional exhaustion.

**Table 4:** Regression results (standardized regression coefficients) predicting the outcomes (*N =* 306).

Effect	*β*	*SE*	*t*	*p*	LLCI	ULCI
Job insecurity to challenge appraisals	0.196	0.056	3.485	Sig	0.0853	0.3067
Challenge appraisals on job satisfaction	–0.067	0.058	–1.158	ns	–0.1125	0.1125
Direct effect of job insecurity on job satisfaction	–0.036	0.058	–0.622	ns	–0.1512	0.0785
Indirect effect of job insecurity on job satisfaction via challenge appraisals	–0.013	0.012	–	ns	–0.0439	0.0057
R-squared mediation effect size	0.001	0.002	–	ns	–0.0010	0.0098
Challenge appraisals on emotional exhaustion	0.082	0.056	1.458	ns	0.1457	–0.0288
Direct effect of job insecurity on emotional exhaustion	0.226	0.056	3.996	Sig	0.1149	0.3378
Indirect effect of job insecurity on emotional exhaustion via challenge appraisals	0.016	0.013	–	ns	–0.0050	0.0495
R-squared mediation effect size	0.009	0.006	–	ns	0.0003	0.0287

*R*^2^ = 0.03; *F*_(1,304)_ = 7.28, *p* < 0.0006						


*p < 0.05, p < 0.01, p < 0.001.*

## Discussion

The major goal of this research was to explore the extent to which hindrance and challenge appraisals of job insecurity mediate the association between job insecurity and well-being related outcomes. The comparison of the mediation effects of cognitive appraisals of job insecurity showed that only 1 out of 4 mediation effects were significant which was not highly strong. Consequently, sufficient evidence was not found to state that cognitive appraisals of job insecurity significantly mediate the association between job insecurity and well-being related outcomes. The only significant mediation path was the job insecurity-hindrance appraisal- emotional exhaustion, showing that a hindrance appraisal of job insecurity mediates the link between job insecurity and emotional exhaustion. This finding is explained by cognitive appraisal theory (Lazarus and Folkman, 1984). According to this theory, a hindrance appraisal of the threat (e.g., job loss) forms a negative anticipation toward how harmful the threat will be. This negative anticipation can undermine or inhibit the coping ability of employees to deal/sustain such threat as they may think that they do not have sufficient ability or means to restrain such stressor. As such, employees with this negative anticipation are expected to report more strains in terms of emotional exhaustion (Sadeghi Vazin et al., 2014). A supplementary explanation for this finding comes from COR theory (Hobfoll, 1989). According to this theory a threatening stressor such as job insecurity undermining the personal resources (i.e., self-efficacy: the perceived ability to overcome job uncertainty; hope: the positive anticipation to find a secure job) or conditional resources (i.e., financial security: the perceived inability to balance their life expenditures) may reduce the sustainability of employees to deal with the threat. A hindrance appraisal of this threat may consume even further the resources of the employees and result in the amplification of the job insecurity-emotional exhaustion association.

Most (3 out of 4) mediation paths were not significant. One of the non-significant effects are related to the mediating role of hindrance appraisals of job insecurity and two to the mediating role of challenge appraisals of job insecurity. The non-significant effects might be due to the following reasons. Hindrance appraisals did not mediate the association between job insecurity and job satisfaction. This might be related to the employment situation of the Iranian participants. As demographic information shows, 79.7% of the participants had a temporary contract. This high rate of temporary contracts in our sample may explain the lack of mediation in the aforementioned association. Employees with temporary contracts are less likely to perceive job insecurity as a breach of their psychological contract with the employer, resulting in less negative reactions (De Cuyper and De Witte, 2006). One reason could be that temporary employees might expect to receive less job security compared to permanent employees (De Cuyper et al., 2010). It means that the lack of job security, as a breach, for the temporary employees might not be as threatening as it might be for the permanent employees. As such, employees are less likely to appraise the breach of psychological contract (e.g., job insecurity) as a hindrance, resulting in no mediation effect. Another reason for not finding a mediation role for hindrance appraisals of job insecurity between job insecurity and job satisfaction might be related to the type of job insecurity was measured. In this respect, researchers claim that quantitative job insecurity has a stronger association with health-related outcomes (e.g., emotional exhaustion) than qualitative job insecurity, whereas qualitative job insecurity has a stronger negative association with job attitudes (e.g., job satisfaction) than quantitative job insecurity (Hellgren et al., 1999). As such, a hindrance appraisal of quantitative job insecurity might have a more detrimental impact in the job insecurity-emotional exhaustion rather than in the job insecurity-job satisfaction association, as found.

No confirmation was found for the mediating role of challenge appraisals of job insecurity in the relationship between job insecurity and outcomes. This shows that what scientists may assume about the protective role of the challenge appraisals in the job insecurity-wellbeing association is not a correct assumption. Since most mediation effects were non-significant, we may conclude that cognitive appraisals of job insecurity do not play a determinant mediating role in the association between job insecurity and outcomes, at least not in the studied sample and country. Our findings suggest that one should distinguish emotional mediators (e.g., social support, affects, and optimism) from cognitive mediators (hindrance vs. challenge appraisals) in the job insecurity-well-being association. According to prior studies, emotional factors may be more likely (e.g., Xanthopoulou et al., 2007) to influence the job insecurity-well-being association than the cognitive factors tested in this study.

The impact of job insecurity on outcomes was replicated in Iran. The results showed that job insecurity is associated with job satisfaction and emotional exhaustion. This replication suggests that the detrimental impact of job insecurity on outcomes is not context specific. The results also showed that hindrance appraisals of job insecurity predict emotional exhaustion. In contrast, challenge appraisals of job insecurity were unrelated to both outcomes. We may thus conclude that hindrance appraisals of job insecurity are more likely to provoke negative outcomes than challenge appraisals. This is consistent with the propositions of cognitive appraisal theory (Lazarus and Folkman, 1984). Accordingly, a perceived hindrance (i.e., job insecurity) is assumed to stimulate a hindrance appraisal. A hindrance appraisal of a stressor is anticipated to result in more various negative outcomes because such appraisals intensify negative feelings of concern and uncertainty. As such, employees may be driven to overestimate the adverse aspects of a threat compared to possible positive aspects. These negative feelings may reduce the personal resources of individuals (e.g., self-efficacy, hope) and produce strains. Challenge appraisals were not associated with the outcomes. We may conclude that this might be because of the fully negative nature of job insecurity in the perceptions of employees. The perception of job insecurity as a hindrance may make greater imbalances in the loss-gain ratio of resources of an individual. In contrast, the perception of job insecurity as a challenge may make fewer imbalances in the same ratio of an individual. The greater perceived imbalance may be more likely to influence the outcomes than the fewer perceived imbalance.

### Suggestions for Future Research

An interesting question for future studies is: what makes an employee appraise job insecurity as a hindrance or as a challenge? Lazarus and Folkman (1984) argued that the cognitive evaluation of a stressor affects not only how stressed you feel, but also what coping strategies you choose, adjust or deal, to overcome a stressor. In doing so, various factors may influence the cognitive evaluation of job insecurity. One might be related to the type of stressor. Stressors that are perceived to have the potential for *rewards* (e.g., praise and recognition), *growth* (e.g., learning new things), and *mastery* (e.g., reaching for a better position) are more likely to be appraised as a challenge; whereas those that are perceived to threaten one’s well-being by frustrating goal attainment and personal development are more likely to be appraised as a hindrance (Lazarus and Folkman, 1984; Skinner and Brewer, 2002; Storch et al., 2007; Webster et al., 2011). For example, job insecurity as a work-related concern has the potential to involve employees in professional development and financial rewards by seeking for an alternative secure job (a challenge appraisal of job insecurity), but also has the potential to demotivate employees to seek for new job opportunities because of the unpredictability of job demands and role complexity of a new job (a hindrance appraisal of job insecurity). The concentration of an employee on the negative (i.e., overestimation of negative impact) or positive (i.e., underestimation of negative impacts) sides of a threat may lead to a hindrance or a challenge appraisal of that threat, respectively. A second factor that may influence the cognitive evaluation of individuals of a threat relates to the level of personal resources. Based on cognitive appraisal theory (Lazarus and Folkman, 1984) and conservation of resources theory (Hobfoll, 1989), individuals who are low in personal resources might be more vulnerable compared to those who are high (Weiss et al., 1999). According to these theories individuals with a lower level of personal resources are more likely to appraise job insecurity as a hindrance rather than a challenge. Future studies may want to examine this hypothesis. A third factor might be associated with the job opportunities or different social security system of a given country. These are so-called societal resources (Senterfitt et al., 2013). Employees of countries with a strong social security system and high job opportunities are probably less likely to appraise job insecurity as a hindrance. They are aware that if they lose their job, they can still be financially supported by their government until they find a new job. Note that this study did not include the possible effects of the societal resources. Future studies may want to test the effect of these and similar societal resources in samples comprising a larger set of countries.

### Strengths and Limitations

The present study contributes to the job insecurity literature in several ways. First, it examines whether the job insecurity-well-being relationship depends on cognitive appraisals of employees of job insecurity. As such, some evidence was found for hindrance appraisals of job insecurity as mediator of the job insecurity-emotional exhaustion association. This finding strengthens the propositions of cognitive appraisal theory. No evidence, however, was found for challenge appraisals of job insecurity as mediator of the job insecurity-well-being relationship. Second, despite the differences in the culture, economic systems, and welfare regimes of Iran, this study replicates the negative associations of job insecurity with two core well-being outcomes, job satisfaction, and emotional exhaustion. Besides, organizational managers may use these findings to design supportive and training programs that help employees to reduce their detrimental appraisal of job insecurity. For example, they can provide employees with enough information of the organizational resources and supports available (Huang et al., 2014), talking to them about the negative consequences of their hindrance appraisals and the way they can turn them into less detrimental appraisals using a clear, realistic, and pragmatic communication program, along with the participation of employees (suggested by Vander Elst et al., 2010a, 2014a), and talking about societal resources available (e.g., social support programs, job loss insurance, bank loans) where they work.

There are also several limitations related to this research that may have affected our conclusions. First, the findings were established based on a cross-sectional research design, which does not allow to study of mediating effects over time. Job insecurity is a phenomenon which is influenced by social and economic shocks (e.g., Setayesh and Mackey, 2016). Studying mediating effects over time may show differences in how cognitive mediators influence the association between job insecurity and wellbeing (Vander Elst et al., 2014a; Piccoli and De Witte, 2015). Future research may apply a longitudinal research design to test the short and long term impacts of job insecurity on these outcomes and to test the influence of both appraisals in the job insecurity-well-being link over time. A second possible limitation concerns the characteristics of the sample: women and white-collar workers were over-represented in comparison to men and blue-collar workers. This selection of workers might limit the generalizability of our findings (e.g., De Witte and Näswall, 2003).

## Conclusion

This research explored that cognitive appraisals of job insecurity hardly play a mediating role in the job insecurity-well-being association. However, when employees appraise job insecurity as a hindrance stressor, job insecurity is more likely to be detrimental and to provoke negative responses. As such, employees hindered by job insecurity are more likely to be emotionally exhausted by perceived job insecurity. Challenge appraisals of job insecurity did not show to have the expected protective role. The replication of the results in Iran, along with same results in other countries, suggested that the detrimental impact of job insecurity on key aspects of well-being (e.g., job satisfaction and emotional exhaustion) is not country-specific.

## Ethics Statement

This study was approved in the Ethical committee of Shahid Beheshti Medical University of Tehran, Iran.

## Author Contributions

MC conducted the research, analyzed the data, and wrote the manuscript.

## Conflict of Interest Statement

The author declares that the research was conducted in the absence of any commercial or financial relationships that could be construed as a potential conflict of interest.
